# High rates of culture conversion and low loss to follow-up in MDR-TB patients managed at Regional Referral Hospitals in Uganda

**DOI:** 10.1186/s12879-021-06743-y

**Published:** 2021-10-12

**Authors:** Mbonye Kayitale Martin, Otuba John Paul, Riese Sara, Alima Hilary, Mugabe Frank, Muhwezi K. Augustin, Turyahabwe Stavia, Wandera Christopher, Tisna Veldhuijzen van Zanten, Tugume Gladys

**Affiliations:** 1 Present Address: Department of Population Studies, School of Statistics and Planning, School of Statistics and Planning, College of Business and Management Sciences, Makerere University, P.O. Box 7062, Kampala, Uganda; 2 University Research Co., LLC, Kampala, Uganda; 3 University Research Co., LLC, Washington, DC USA; 4 National TB and Leprosy Control Division, Ministry of Health, Kampala, Uganda

**Keywords:** Multi-drug resistant tuberculosis, Regional referral hospitals, Uganda

## Abstract

**Background:**

Multi-drug resistant—tuberculosis (MDR-TB) is an emerging public health concern in Uganda. Prior to 2013, MDR-TB treatment in Uganda was only provided at the national referral hospital and two private-not-for profit clinics. From 2013, it was scaled up to seven regional referral hospitals (RRH). The aim of this study was to measure interim (6 months) treatment outcomes among the first cohort of patients started on MDR-TB treatment at the RRH in Uganda.

**Methods:**

This was a cross-sectional study in which a descriptive analysis of data collected retrospectively on a cohort of 69 patients started on MDR-TB treatment at six of the seven RRH between 1st April 2013 and 30th June 2014 and had been on treatment for at least 9 months was conducted.

**Results:**

Of the 69 patients, 21 (30.4%) were female, 39 (56.5%) HIV-negative, 30 (43.5%) resistant to both isoniazid and rifampicin and 57 (82.6%) category 1 or 2 drug susceptible TB treatment failures. Median age at start of treatment was 35 years (Interquartile range (IQR): 27–45), median time-to-treatment initiation was 27.5 (IQR: 6–89) days and of the 30 HIV-positive patients, 27 (90.0%) were on anti-retroviral treatment with a median CD4 count of 206 cells/microliter of blood (IQR: 113–364.5). Within 6 months of treatment, 59 (85.5%) patients culture converted, of which 45 (65.2%) converted by the second month and the other 14 (20.3%) by the sixth month; one (1.5%) did not culture convert; three (4.4%) died; and six (8.8%) were lost-to-follow up. Fifty (76.8%) patients experienced at least one drug adverse event, while 40 (67.8%) gained weight. Mean weight gained was 4.7 (standard deviation: 3.2) kilograms.

**Conclusions:**

Despite MDR-TB treatment initiation delays, most patients had favourable interim treatment outcomes with majority culture converting early and very few getting lost to follow-up. These encouraging interim outcomes indicate the potential for success of a scale-up of MDR-TB treatment to RRH.

## Background

The diagnosis and treatment of Multidrug Resistant—Tuberculosis (MDR-TB) which is resistant to two of the most efficacious first line anti-TB medicines (Isoniazid and Rifampicin) remains a big challenge globally [1]. The World Health Organization (WHO) estimates that in 2017, there were 558,000 new cases of MDR-TB, and of these only 160,684 cases were diagnosed, with treatment success rate as low as 55% [2]. The low treatment success rate can partly be attributed to the long duration of treatment, treatment adherence difficulties, and common adverse events [3, 4]. Although WHO has developed guidelines for diagnosis and treatment of MDR-TB [3, 5–7], access to MDR-TB treatment in high burden countries has only been increasing slowly [8], indicating challenges in implementation of the guidelines [9]. Until recently, the limited availability of second line drugs for the treatment of MDR-TB has been a major barrier to treatment access [10]. The gap in MDR-TB treatment coverage globally has also been attributed to inadequacies within the health system, including but not limited to lack of skilled human resources for health, lack of MDR-TB medicines and other logistics for MDR-TB patient management, inadequate infrastructure for in-patient care and suboptimal TB infection control practices, lack of funding for patient social support and limited access to laboratory and other monitoring tests [3].

As of 2014, Uganda was one of the 22 high burden TB countries in the world with approximately 44,187 incident TB cases (161/100,000). Over half of TB patients were co-infected with Human Immuno-Deficiency Virus (HIV), and the country suffers a high HIV burden with a national prevalence of 7.3% [11]. Approximately 1.4% of new sputum positive cases and 12.1% of previously treated TB cases were MDR-TB, which translated into approximately 1,100 new MDR-TB cases in 2014 alone [3]. However, the MDR-TB case notification rate was notably much higher than the MDR-TB treatment enrollment rate [12], indicating gaps in access to MDR-TB treatment. It was therefore necessary to further decentralise MDR-TB services in order to improve access to MDR-TB treatment. Until 2012, MDR-TB treatment was only being provided at the National Referral Hospital (NRH) and two Médecins Sans Frontières (MSF) run clinics at Arua Regional Referral Hospital (RRH) and Kitgum government general hospital [13]. In 2012, the National TB and Leprosy Programme (NTLP) began mobilizing partner support to further decentralize treatment of drug resistant TB (DR-TB) to other RRHs to increase coverage and make treatment more accessible.

In collaboration with NTLP, the United States Agency for International Development (USAID) funded Strengthening Uganda’s System for Treating AIDS Nationally (SUSTAIN) project to build capacity for management of DR-TB at seven RRH in 2013. A phased approach to scale up ambulatory MDR-TB care to the seven RRH with the highest incidences of MDR-TB was adopted. Scale-up included training of multi-disciplinary teams at each RRH to initiate and manage MDR-TB treatment, dissemination of guidelines for management of MDR-TB, and facilitation of follow-up of patients and their household contacts. While the WHO recommends ambulatory MDR-TB models [3, 14], in Uganda, the NTLP recommends a mixed model of care that combines both ambulatory and hospitalisation approaches [15].

To understand if the scale up of MDR-TB treatment to RRH was successful in ensuring favorable interim treatment outcomes (sputum culture conversion), we conducted a cross sectional study to assess the early treatment outcomes for the first cohort of MDR-TB patients started on MDR-TB treatment at seven RRH. To our knowledge, this is the first analysis of this kind in the country.

## Methods

### Study type, site and population

**Fig. 1:**
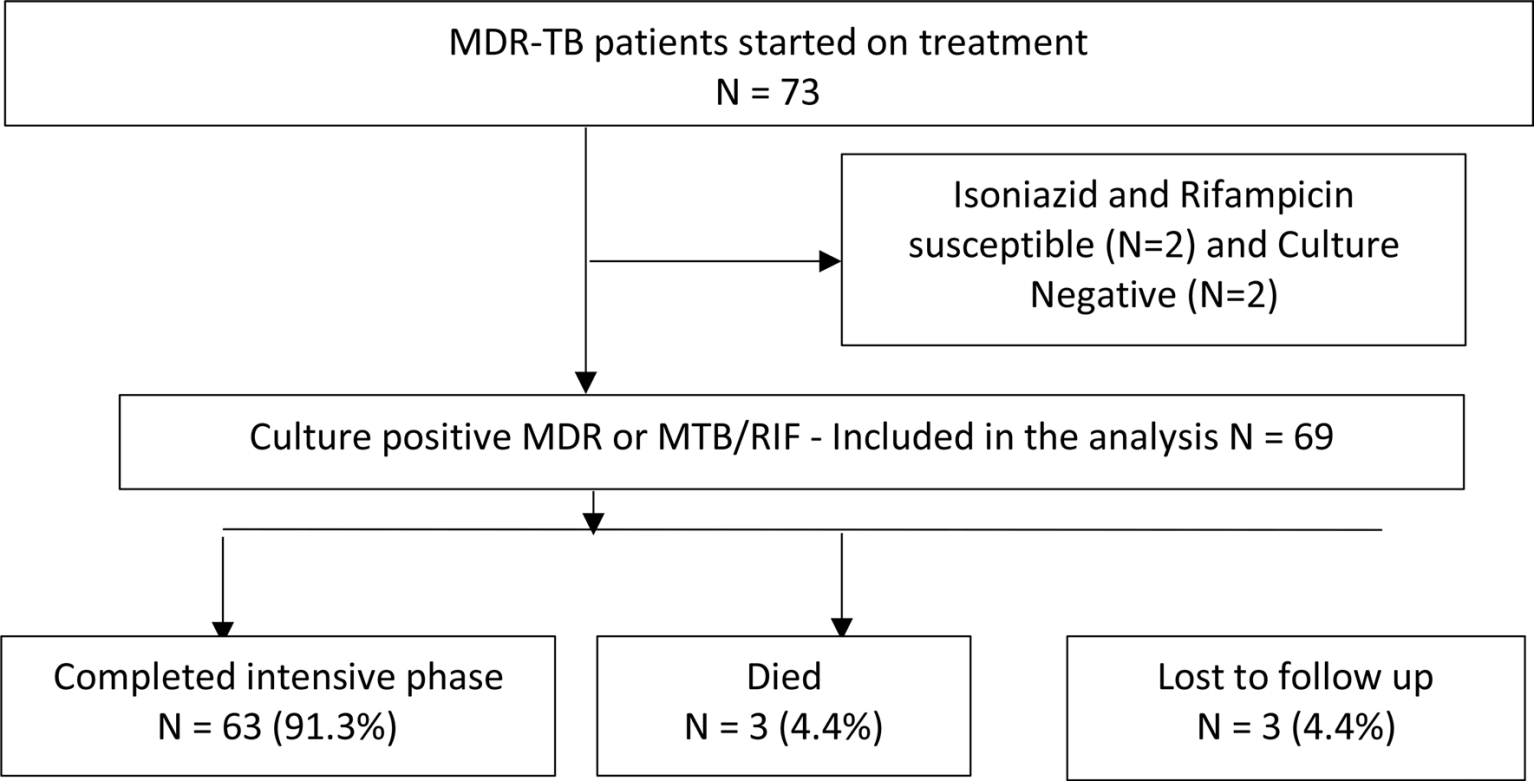
Flow chart for patients initiated on MDR-TB treatment from April 2013 to June 2014

The inclusion criteria for patients in this cross-sectional study (Fig. 1) were:

- Started on MDR-TB treatment from 1st April 2013 through 30th June 2014; and
- Culture positive MDR or *Mycobacterium tuberculosis* and resistance to Rifampcin (MTB/RIF); and
- On treatment for at least 9 months; and
- Have available 6 -month culture results

By the time of this study, six of the seven RRHs had started patients on MDR-TB treatment. With these criteria, 69 patients at the six RRHs were included in the study.

### Mycobacteria culture and drug susceptibility testing

Prior to the study period, sputum samples from all MDR-TB patients at the six RRHs had culture and Drug Sensitivity Testing (DST) done at the National TB Reference Laboratory (NTRL). Results were categorised according to the Uganda national guidelines for programmatic management of drug resistant TB [16, 17] as follows:

1. *Category 1 failure*: patients who had received first-line drug for treatment of pulmonary bacteriologically confirmed TB and for whom treatment had failed (sputum smear-positive) at 5 months or later during the course of treatment.
2. *Category 2 failure*: patients who had received first-line drug for treatment of pulmonary bacteriologically confirmed TB and for whom treatment had failed (sputum smear-positive) at 3 months or later during the course of treatment.
3. *Relapse*: patients who had been previously treated for TB and whose most recent treatment outcome was *Cured* or *Treatment completed*, and who were subsequently diagnosed with a recurrent episode of TB (either a true relapse or a new episode of TB caused by reinfection).
4. *Treatment after loss to follow-up*: patients who had previously been treated for TB and were declared *Lost to follow-up* at the end of the most recent course of treatment (this category was previously known as *treatment after default*).
5. *New*: patients who had received no (or less than 1 month) of anti-TB treatment.

### MDR-TB regimen and treatment follow-up

Baseline history, physical examination, chest radiography, haematology and chemistry tests, were conducted at the RRHs before initiating a patient on MDR-TB treatment. Multidisciplinary teams conducted pre-treatment preparation and counselling of the patient, after which a standardised MDR-TB regimen was initiated in accordance with Uganda National MDR-TB guidelines [15, 17].

All patients were first initiated on the hospitalisation model of care for a period of 2 months after which they were transitioned to ambulatory model of care. Patients whose treatment response was not progressing well as determined by the physicians were kept on hospitalization model of care beyond 2 months. For the ambulatory model of care, the patient was transferred to a peripheral health facility nearest to their home to receive their treatment. At the peripheral health facility, the patients would continue receiving ambulatory Directly Observed Treatment (DOT), adherence monitoring, and psychosocial support. Before transferring a patient to the peripheral health facility, a team of health workers from the RRH visited the health facility, assessed it and trained staff in MDR-TB case management. The initiating RRH conducted monthly visits to the peripheral health facilities and provided supervision and mentorship to health workers. A monthly supply of MDR-TB and ancillary medicines as well as personal protective equipment for TB infection control were delivered to the health facility.

In the ambulatory model of care, health workers conducted home visits to provide relevant health education to the patient and family, assess for adherence to infection control practices, screen household contacts for TB symptoms and collect sputum samples for GeneXpert^®^ MTB/RIF testing.

The treatments for all MDR-TB patients involved two phases: (1) the intensive phase and (2) continuation phase. During the intensive phase, the patient received an injectable agent (kanamycin) until they culture converted. A culture was done before the start of treatment and subsequently every month. Culture conversion was defined as two consecutive negative cultures, thirty days apart after commencing treatment. Following culture conversion, the patient continued to receive the injectable agent for an additional 4 months, after which they entered into the continuation phase. In the continuation phase, the injectable agent was discontinued with the patient continuing on their initial treatment regimen until the eighteenth (18th) month. The 18th month is the minimum time point at which a patient was expected to complete the entire treatment phase [15]. Every MDR-TB patient was also counselled and tested for HIV and those found to be MDR-TB/HIV co-infected were linked to integrated TB/HIV services and initiated on an Anti-Retroviral Therapy (ART) regimen containing a backbone of either Zidovudine/Lamivudine or Tenofovir/Emtricitabine and a tail of Efavirenz or Nevirapine within 2 months of MDR-TB treatment initiation, regardless of CD4 + count [18].

Every month, patients received a comprehensive monthly review in which sputum samples were collected for monthly follow-up cultures, kidney and liver functions were analysed, blood samples were collected and delivered to a private laboratory for thyroid function tests and patients were assessed for hearing impairment. For co-infected MDR-TB/HIV patients, all services integrated MDR-TB and HIV care, such as screening and management of opportunistic infections, response to ART, Immune Reconstitution Inflammatory Syndrome, repeat CD4 + and viral load testing after 6 months according to WHO HIV treatment guidelines [18]. Also, during the monthly review, the patients received nutritional assessment, counselling and support. They were also monitored for drug adverse events and managed with dose adjustments, substitution of the offending drug, treatment withdrawal and/or by reassurance. Adverse events were either self-reported or clinically confirmed through conducting the various possible physical and laboratory examinations.

### Six months interim treatment outcomes

The 6 months interim treatment outcome was binary and categorized as favourable or unfavourable as per the national guidelines [15, 17]. A patient with a favourable treatment outcome was one who culture converted from positive to negative [19].

A patient with an unfavourable treatment outcome included one who:

1. remained culture positive at the end of 6 months of MDR-TB treatment; or
2. died for any reason during the course of 6 months of treatment; or
3. by the end of the 6 months of the treatment period, had had treatment interrupted for two or more consecutive months without a medical reason by the end of the 6 months of the treatment period; or
4. by the end of the 6 month had no treatment outcome assigned, including patients transferred out to another treatment unit or whose treatment outcome is unknown.

### Data collection and analysis

The following data on patient background characteristics were collected through a retrospective record review: Demographic data (age and sex), patient’s medical history at the start of MDR-TB treatment (history of the outcome of the first-line TB treatment, HIV status, ART and CD4 + count status for HIV positive patients) and MDR-TB treatment status (time to treatment initiation, MDR-TB treatment regimen, drug resistance profile, treatment model, monthly treatment weights, occurrence of treatment adverse events, monthly smear and culture monitoring results). A descriptive analysis of the data was conducted using STATA statistical software (version 14.0).

## Results

### Demographic and clinical characteristics

Characteristics of the 69 patients at the start of treatment are shown in Table 1. A total of 30 (43.5%) patients were resistant to both isoniazid and rifampicin on culture based DST and 39 (56.5%) were resistant to at least rifampcin based on GeneXpert^®^ result.

**Table 1:** Characteristics of MDR-TB patients at the start of treatment

Variable	All patients (n = 69) N (%)
Demographics	
Age (in years)	
Median (IQR)	35 (27–45)
Sex	
Female	21 (30.4%)
Patients’ medical history at the start of treatment	
TB treatment history/patient type	
Category 1 failure	20 (29.0%)
Category 2 failure	37 (53.6%)
New	5 (7.3%)
Relapse	1 (1.5%)
Treatment after loss-to-follow up/defaulter	6 (8.7%)
Drug resistance profile	
Resistant to both isoniazid and rifampicin on culture based DST	30 (43.5%)
MTB/RIF based on GeneXpert^®^ result	39 (56.5%)
HIV status	
Positive**	30 (44.5%)
ART status of HIV positive cases (N = 30)	
On ART	27 (90%)
CD4 Cell count/microliter of blood (N = 30)	
CD4 cell count is known	28 (93.3%)
CD4 cell count is not known	2 (6.7%)
Median CD4 cell count (IQR)	206 (113–364.5)

**All HIV positive patients received ART and Cotrimoxazole prophylaxis; IQR is the Interquartile Range

### Interim treatment outcomes

The median time to treatment initiation following diagnosis of the patient was 27.5 (Range 6–89) days. By the end of the 6 months, 48 (69.6%) of the patients had gone through the hospitalization and ambulatory models of care, while 21 (30.4%) had experienced only hospitalization model of care. A total of 50 (72.5%) of the patients gained weight during the treatment period, with the mean weight gain being 4.7 kg, 18 (26.1%) lost weight with the mean weight loss being 2.8 kg while the rest (1.4%) neither gained nor lost any weight.

Table 2 shows the 6 months interim treatment outcomes and the period to culture conversion. Most (85.5%) patients culture converted within a period of 6 months with the median time to culture conversion was 1 month (Inter Quartile Range 1–2 months).

**Table 2:** Six-months interim treatment outcomes and culture conversion time

Variable	All patients (n = 69) N (%)
Six months interim treatment outcomes	
Favourable outcome	
Culture converted	59 (85.5%)
Unfavourable outcome	
Remained culture positive	1 (1.4%)
Culture unknown	3 (4.3%)
Died	3 (4.3%)
Lost-to-follow up	3 (4.3%)
Culture conversion time (by the sixth month of treatment)	
By 2 months	45 (76.3%)
Between 3 and 6 months	14 (23.7%)
Median time to culture conversion (months)	1.0 (1.0–2.0)

**Fig. 2:**
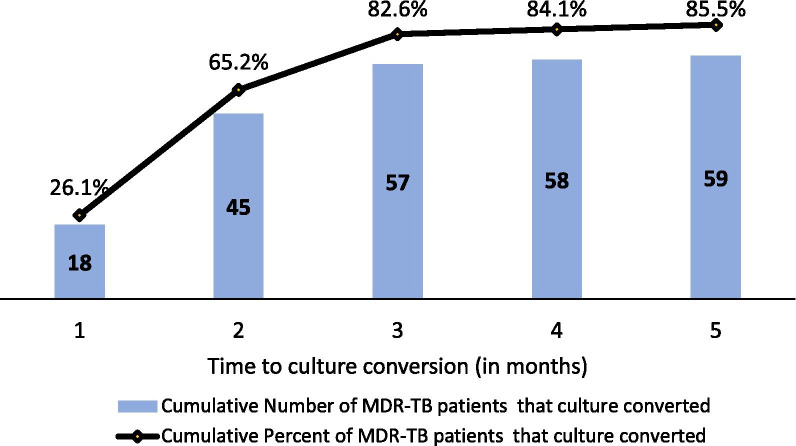
Time to sputum culture conversion among 59 patients with favorable interim treatment outcomes

Figure 2 shows that, cumulatively, majority (65.2%) of the 59 patients with a favourable treatment interim treatment outcome culture converted by the end of the first 2 months following onset of treatment.

### Interim treatment outcomes by type/category of patient

Most patients who had been either Category 1 or 2 failure at the start of MDR-TB treatment had a favourable treatment outcome by the end of the 6 months of MDR-TB treatment (Table 3). The one patient who had relapsed on the first TB treatment was still positive at the end of the 6 months of treatment on MDR-TB.

**Table 3:** Interim 6-months treatment outcomes by type of patient

Patient type at start of MDR-TB treatment	Number of patients	Number (Percent) of patients with favourable treatment outcome
Category 1 failure	20	20 (100%)
Category 2 failure	37	31 (83.8%)
*All failures (Categories 1 and 2)*	*57*	*58 (87.8%)*
Loss to follow up	6	4 (66.7%)
New	5	4 (80.0%)
Relapse	1	0 (0.0%)

The Italic values are a total of Category 1 and Category 2 failures

### Adverse events

A total of 53 (76.8%) patients experienced some form of adverse events, with the mean number of adverse events experienced per patient being 1.8, and some of the patients experiencing as many as up to 7 adverse events during the 6 months treatment period. Table 4 shows the specific MDR-TB treatment associated adverse events that were experienced by patients. Hearing impairment was the most prevalent adverse event, occurring in 30.4% of the patients with a reported adverse treatment event. With exception of burning pains in the lower limbs, adverse events were managed symptomatically without need for treatment discontinuation.

**Table 4:** Frequency of adverse events and how they were resolved (N = 53)

Adverse event	Reaction n (%)	How adverse events were resolved		
		Managed clinically	Regimen changed	Other^b^
Nausea/vomiting	14 (20.3%)	14 (100%)		
Headache	15 (21.7%)	15 (100%)		
Diarrhea	2 (2.9%)	2 (100%)		
Burning pains in the lower limbs	20 (29.0%)	18 (90%)	1 (5%)	1 (5%)
Arthralgia	12 (17.4%)	12 (100%)		
Seizures	1 (1.4%)	1 (100%)		
Rash	1 (1.4%)	1 (100%)		
Psychiatric manifestations^a^	9 (13.0%)	9 (100%)		
Hypothyroidism	2 (2.9%)	2 (100%)		
Visual disturbances (Reduced visual acuity)	10 (14.5%)	10 (100%)		
Hearing disturbance (Bilateral mild through to profound hearing loss)	21 (30.4%)	21 (100%)		
Dizziness	13 (18.8%)	13 (100%)		

^a^Psychiatric manifestations were spectrum of mental disorders as described by WHO including among others bad dreams, convulsions, suicidal thoughts, insomnia, headaches, anxiety, nervousness, memory or mood changes

^b^“Other” referred to the other ways the patient with an adverse event was managed other than regimen change or clinical management. In this case, the one patient was just reassured by the clinician that the adverse event would eventually self-resolve and encouraged to continue taking their medication for MDR-TB

## Discussion

This manuscript reports results of the first efforts by government of Uganda with support of development partners to scale up MDR-TB treatment in public health facilities in Uganda from the NRH to the next tier of the national health care system, that is the RRH.

Overall, 85.5% of the patients had a favourable treatment outcome (culture converted) within 6 months of onset of MDR-TB treatment. Although some results have been documented in South Africa, Lesotho, Nigeria and Tanzania [20–24], there is generally paucity of literature on interim treatment outcomes among patients on MDR-TB treatment on the African continent [25, 26]. The high sputum culture conversion rate (85.5%) was comparable (average of 70%) to results from 5 other countries: Estonia, Latvia, Peru, the Philippines, and the Russian Federation [27].

The 4.4% loss to follow up rate observed in this patient population is low compared to previous community based cohorts reported in Peru and South Africa [28, 29]. We postulate that the combination hospitalization and ambulatory MDR-TB treatment model contributed to the low loss to follow up by ensuring that patients received treatment easily either during hospitalization or had to travel only a short distance to receive treatment at the peripheral health facilities. During treatment, patients were also able to interact more with trained healthworkers who re-enforced adherence and retention in care. The decentralisation of MDR-TB management to RRH, coupled with utilisation of peripheral health facilities nearest to patient homes for daily DOT broadened MDR-TB treatment coverage and access and minimised loss to follow up.

Generally, mortality at the end of the 6 months of treatment was low (4.4%), occurring in only three patients—all HIV co-infected and of whom two were not on ART. This high mortality among HIV co-infected patients compares with observations in previous studies [18]. In this study, mortality occurred early-within 4 weeks of MDR-TB treatment initiation, underscoring the need for expedited treatment initiation among MDR-TB patients. A meta-analysis by Wells et al. reported death among MDR-TB patients on treatment occuring within 4–8 weeks of MDR-TB diagnosis and also demonstrated higher mortality rates among HIV co-infected patients, and even among patients on ART [30].

We observed a high number of patients (76.8%) that experienced some form of adverse drug events. Adverse events such as hearing impairment were majorly due to the MDR-TB medicines in the treatment regimen, like Kanamycin. Routine audiometry and Kanamycin substitution may therefore benefit the patient. Other adverse events like peripheral neuropathy are known pyrazinamide, ethionamide and cycloserine associated events, potentiated by HIV related peripheral neuropathies. We postulate that the high frequency of adverse events observed were probably associated with poor clinical condition of the patients and existing MDR-TB/HIV coinfection. Most of these adverse events were symptomatic, meaning that as a result of laboratory resource constraints, patients missed on other key laboratory procedures to test for additional adverse events. There is need for further research to elucidate predictors of the high prevalence of adverse events among MDR-TB patients on treatment.

Until 2012, MDR-TB treatment in Uganda was only provided at the NRH and two MSF run MDR-TB clinics at Arua RRH and Kitgum government general hospital [13]. At that time, RRH only had capacity to do surveillance and referral to the three sites for treatment. Patients diagnosed at RRH had challenges accessing treatment due to long distances to the centers, lack of social/family support and lack of food packages during the mandatory hospitalization phase of treatment. The SUSTAIN project supported 7 RRH’s MDR-TB treatment sites to address these access and coverage challenges since 2013, and early treatment outcomes in this study indicate the potential for success of a scale up of MDR-TB treatment to RRH.

Six aspects of SUSTAIN’s approach to MDR-TB treatment scale-up should be considered for further scale-up to other health facilities; First, utilization of an effective tailor-made and simpler standardized MDR-TB treatment regimen training, drug forecasting and management of drugs adverse events; Second, the existing DOT programs at peripheral health facilities which were supervised by the decentralized RRH MDR-TB sites which enabled individual patients to access daily medication closer to their homes; Third, the monthly reviews of patients by the multidisciplinary teams which ensured prompt identification and management of adverse events minimized treatment interruptions and ultimately loss to follow-up; Fourth, the monthly home visits and psychosocial support, adherence and nutritional assessment counselling and support provide by the RRH multidisciplinary team; Fifth, the integrated MDR-TB/HIV services run by the MDR-TB clinics which reduce patient waiting time and also provide an opportunity to the multidisciplinary teams to screen for drug adverse events and interactions and provide appropriate management; and finally, the ambulatory care coupled with TB infection control measures and practices at the RRH during the early days of hospitalization may contribute to prevention of reinfection with resistant strains and hence the positive treatment outcomes observed.

The feasibility of ambulatory MDR-TB models of care is still debated [22, 23, 31]. Equally, community based cohorts have demonstrated challenges with patient adherence, leading to high loss to follow-up rates [28, 29]. Our findings provide insights into the early treatment outcomes from a mixed model of MDR-TB care in resource constrained settings. Adopting a mixed model of care that starts with hospitalization may provide the best opportunity to identify patients at higher risk of loss to follow-up before transitioning to ambulatory care.

The study was not without limitations. The source of data was patient registers at the health facilities. Using data from patient medical note books would have enabled to obtain more detailed data on results of clinical examination to further describe patient profiles. The clinics were also not capturing data on patient’s height and as thus, it was not possible to measure important anthropometric indicators such as Body Mass Index (BMI) to better explain nutritional status of patients.

## Conclusions

This treatment model, combining hospitalization in the first months when the patient was very sick and starting treatment and an ambulatory model later when the patient was familiar with treatment and potentially feeling better ensured a sustained patient-trained health worker interaction. Findings of this study indicate that, despite MDR-TB treatment initiation delays, most patients had favourable interim treatment outcomes, with majority culture converting early and very few dying or getting lost to follow-up. A high proportion of patients experienced some form of adverse drug events with most adverse drug events managed clinically without having to change the treatment regimen. Although this study was not designed to find if a mixed model of care is superior to single dimensional models, these early treatment results point towards early success of the scale-up of mixed-model MDR-TB programme in resource constrained public health facilities in Uganda. We would like to believe that this model of MDR-TB care significantly shortened the long distances patients travelled to access treatment at NRH and 2 MSF sites in Uganda, and also eventually enabled patients that would otherwise have missed an opportunity to receive treatment to access it. Qualitative studies will be necessary to provide insights into health workers’ perspectives and patient’s experiences regarding the mixed model of MDR-TB care in Uganda.

## Data Availability

The dataset used during this study is available and can be shared upon request from the corresponding author.

## Abbreviations

ART: Anti-retroviral therapy
CHC: Center for health services
DOT: Directly observed treatment
DST: Drug susceptibility test
HIV: Human immuno-deficiency virus
LPA: Line probe assays
MDR: Multi-drug resistance tuberculosis
MSF: Médecins Sans Frontières
NTLP: National TB and leprosy program
NTRL: National TB reference laboratory
PAS: Para-amino salicylic acid
REC: Research and Ethics Committee
RR: Rifampicin resistance
RRH: Regional Referral Hospital
SD: Standard deviation
SUSTAIN: Strengthening Uganda Health Systems for Treating AIDS Nationally
TB: Tuberculosis
URC: University Research Co., LLC
USAID: United States Agency for International Development
WHO: World Health Organization
